# Clinical Profiles and Patterns of Kidney Disease Progression in C3 Glomerulopathy

**DOI:** 10.34067/KID.0000000000000115

**Published:** 2023-03-30

**Authors:** Fernando Caravaca-Fontán, Teresa Cavero, Montserrat Díaz-Encarnación, Virginia Cabello, Gema Ariceta, Luis F. Quintana, Helena Marco, Xoana Barros, Natalia Ramos, Nuria Rodríguez-Mendiola, Sonia Cruz, Gema Fernández-Juárez, Adela Rodríguez, Ana Pérez de José, Cristina Rabasco, Raquel Rodado, Loreto Fernández, Vanessa Pérez-Gómez, Ana Ávila, Luis Bravo, Natalia Espinosa, Natalia Allende, Maria Dolores Sanchez de la Nieta, Eva Rodríguez, Begoña Rivas, Marta Melgosa, Ana Huerta, Rosa Miquel, Carmen Mon, Gloria Fraga, Alberto de Lorenzo, Juliana Draibe, Fayna González, Amir Shabaka, Maria Esperanza López-Rubio, María Ángeles Fenollosa, Luis Martín-Penagos, Iara Da Silva, Juana Alonso Titos, Santiago Rodríguez de Córdoba, Elena Goicoechea de Jorge, Manuel Praga

**Affiliations:** 1 Department of Nephrology, Instituto de Investigación Hospital 12 de Octubre (imas12), Madrid, Spain; 2 Department of Medicine, Universidad Complutense de Madrid, Madrid, Spain; 3 Department of Nephrology, Hospital Universitario 12 de Octubre, Madrid, Spain; 4 Department of Nephrology, Fundación Puigvert, Barcelona, Spain; 5 Department of Nephrology, Hospital Universitario Virgen del Rocío, Sevilla, Spain; 6 Department of Pediatric Nephrology, Hospital Universitario Vall d’Hebron, Universidad Autónoma de Barcelona, Barcelona, Spain; 7 Department of Nephrology and Renal Transplantation, Hospital Clinic de Barcelona; Department of Medicine, University of Barcelona, IDIBAPS, Barcelona, Spain; 8 Department of Nephrology, Hospital Universitari Germans Trias i Pujol, Badalona, Barcelona, Spain; 9 Current address: Department of Nephrology, Fundación Puigvert, Barcelona, Spain; 10 Department of Nephrology, Hospital Universitario Doctor Josep Trueta, Gerona, Spain; 11 Department of Nephrology, Hospital Universitario Vall d’Hebron, Barcelona, Spain; 12 Department of Nephrology, Hospital Universitario Ramón y Cajal, Madrid, Spain; 13 Department of Nephrology, Hospital Universitario Juan Ramón Jiménez, Huelva, Spain; 14 Department of Nephrology, Hospital Universitario Fundación Alcorcón, Alcorcón, Madrid, Spain; 15 Current address: Department of Nephrology, Hospital Universitario La Paz, Madrid, Spain; 16 Department of Pediatric Nephrology, Hospital Universitario Virgen del Rocío, Sevilla, Spain; 17 Department of Nephrology, Hospital Universitario Gregorio Marañón, Madrid, Spain; 18 Department of Nephrology, Hospital Universitario Reina Sofía, Córdoba, Spain; 19 Department of Nephrology, Hospital Universitario Virgen de la Arrixaca, Murcia, Spain; 20 Department of Nephrology, Complejo Hospitalario de Navarra, Navarra, Spain; 21 Department of Nephrology, Hospital Universitario Fundación Jiménez Díaz, Madrid, Spain; 22 Department of Nephrology, Hospital Universitario Doctor Peset, Valencia, Spain; 23 Department of Nephrology, Hospital Universitario A Coruña, La Coruña, Spain; 24 Pediatric Nephrology Unit, Hospital Universitario Son Espases, Balearic Islands Health Research Institute (IdISBa). Palma de Mallorca, Spain; 25 Department of Nephrology, Hospital Universitario Son Espases, Palma de Mallorca, Spain; 26 Department of Nephrology, Hospital General Universitario de Ciudad Real, Ciudad Real, Spain; 27 Department of Nephrology, Hospital del Mar, Barcelona, Spain; 28 Department of Nephrology, Hospital Universitario La Paz, Madrid, Spain; 29 Department of Pediatric Nephrology, Hospital Universitario La Paz, Madrid, Spain; 30 Department of Nephrology, Hospital Universitario Puerta de Hierro, Madrid, Spain; 31 Department of Nephrology, Hospital Universitario Canarias, Tenerife, Spain; 32 Department of Nephrology, Hospital Universitario Severo Ochoa, Leganés, Madrid, Spain; 33 Department of Pediatric Nephrology, Hospital de la Santa Creu i Sant Pau, Universitat Autònoma de Barcelona, Barcelona, Spain; 34 Department of Nephrology, Hospital Universitario de Getafe, Madrid, Spain; 35 Department of Nephrology, Hospital Universitario de Bellvitge, Barcelona, Spain; 36 Department of Nephrology, Hospital Doctor Negrín, Gran Canaria, Spain; 37 Department of Nephrology, Hospital Universitario Clínico San Carlos, Madrid, Spain; 38 Department of Nephrology, Complejo Hospitalario Universitario de Albacete, Albacete, Spain; 39 Department of Nephrology, Hospital General Universitario de Castellón, Castellón, Spain; 40 Department of Nephrology, Hospital Universitario Marqués de Valdecilla, Santander, Spain; 41 Current address: Department of Nephrology, Hospital Universitari Germans Trias i Pujol, Badalona, Barcelona, Spain; 42 Department of Nephrology, Hospital Regional Universitario Carlos Haya, Málaga, Spain; 43 Centro de Investigaciones Biológicas, Consejo Superior de Investigaciones Científicas, Madrid and Centro de Investigación Biomédica en Red en Enfermedades Raras, Madrid, Spain; 44 Department of Immunology, Universidad Complutense de Madrid, Madrid, Spain

**Keywords:** chronic kidney disease, complement, glomerulonephritis

## Abstract

**Key Points:**

- Kidney survival in C3 glomerulopathy is significantly higher in patients with a disease chronicity score <4 and proteinuria <3.5 g/d, regardless of baseline eGFR.
- A faster eGFR decline in C3 glomerulopathy is associated with higher probability of kidney failure.
- Patients with glomerulopathy with a progressive reduction in proteinuria over time did not reach kidney failure.

**Background:**

C3 glomerulopathy is a rare kidney disease, which makes it difficult to collect large cohorts of patients to better understand its variability. The aims of this study were to describe the clinical profiles and patterns of progression of kidney disease.

**Methods:**

This was a retrospective, observational cohort study. Patients diagnosed with C3 glomerulopathy between 1995 and 2020 were enrolled. Study population was divided into clinical profiles by combining the following predictors: eGFR under/above 30 ml/min per 1.73 m^2^, proteinuria under/above 3.5 g/d, and histologic chronicity score under/above 4. The change in eGFR and proteinuria over time was evaluated in a subgroup with consecutive measurements of eGFR and proteinuria.

**Results:**

One hundred and fifteen patients with a median age of 30 years (interquartile range 19–50) were included. Patients were divided into eight clinical profiles. Kidney survival was significantly higher in patients with a chronicity score <4 and proteinuria <3.5 g/d, both in those presenting with an eGFR under/above 30 ml/min per 1.73 m^2^. The median eGFR slope of patients who reached kidney failure was −6.5 ml/min per 1.73 m^2^ per year (interquartile range −1.6 to −17). Patients who showed a reduction in proteinuria over time did not reach kidney failure. On the basis of the rate of eGFR decline, patients were classified as faster eGFR decline (≥5 ml/min per 1.73 m^2^ per year), slower (<5 ml/min per 1.73 m^2^ per year), and those without decline. A faster eGFR decline was associated with higher probability of kidney failure.

**Conclusions:**

Kidney survival is significantly higher in patients with a chronicity score <4 and proteinuria <3.5 g/d regardless of baseline eGFR, and a faster rate of decline in eGFR is associated with higher probability of kidney failure.

## Graphical abstract

**Figure:**
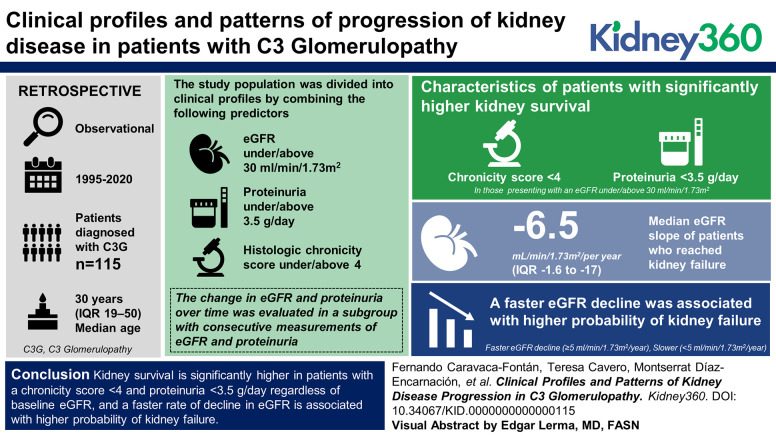

## Introduction

C3 glomerulopathy (C3G) is a highly complex kidney disease caused by alternative complement pathway dysregulation.^1–3^ The estimated incidence of C3G is 1–3 cases per million population per year,^4,5^ which makes it difficult to collect large cohorts of patients.

Cumulative experience from cohort studies has shown that age, eGFR, and proteinuria are among the most important clinical predictors of kidney failure.^5–10^ Furthermore, the changes in proteinuria over time have also been associated with outcomes in C3G.^11^ On the other hand, histologic features of disease activity and, particularly disease chronicity, have been associated with outcomes in C3G, demonstrating that a timely diagnosis is essential to mitigate the extent of kidney damage.^4,10,12^ Despite this, the individual variability in clinical course and patterns of disease progression according to baseline characteristics have rarely been studied in C3G.

After years of research, several new anticomplement agents are currently under investigation.^3^ Preliminary results with the use of avacopan, a selective C5a receptor inhibitor, revealed that the drug attenuated the histologic disease chronicity compared with placebo, although this study failed to achieve the primary end point which was the reduction in disease activity.^13^ In addition, preliminary results with pegcetacoplan, a C3 inhibitor administered through subcutaneous infusion, showed a reduction in proteinuria and stabilization of kidney function.^14^ Finally, preliminary results with iptacopan, an oral selective inhibitor of factor B, showed a proteinuria reduction by 45% in native kidneys, together with a significant reduction in C3 deposits in kidney transplant recipients, and sustained normalization of plasma C3 and creatinine clearance levels, associated with an improvement in eGFR slope.^15^

Pending the definitive results from ongoing trials with these agents, there is a need from a clinical perspective to better describe the patterns of disease progression according to baseline characteristics.^16^ This real-world evidence could help to identify subgroups of patients who could benefit most from these newer anticomplement therapies when they become available.

Hence, the aims of this study were to describe the different clinical profiles and patterns of progression of kidney disease in a multicentric cohort of 115 Spanish patients with C3G.

## Methods

### Study Patients

Patients diagnosed with C3G between 1995 and 2020 in 35 nephrology departments belonging to the Spanish Group for the Study of Glomerular Diseases were enrolled. Both pediatric and adult populations were included. Patients with missing data on baseline kidney function, proteinuria, or outcomes were excluded.

A diagnosis of C3G required C3 staining on immunofluorescence at least two orders of magnitude greater than any immunoglobulin staining.^17^ Patients were considered to have dense deposit disease when highly electron-dense intramembranous deposits were observed on electron microscopy and C3 glomerulonephritis when deposits did not fulfill this criterion.

Baseline and follow-up data were compiled from the medical records as described elsewhere.^12,18^ Data on eGFR, serum albumin, C3 levels, and proteinuria were collected at baseline and after 1, 3, 6, 12, 24, 60 months, and/or last follow-up. Complement genetics were performed as shown in Supplemental Methods. Plasma C3 levels were categorized as under/above 77 mg/dl, on the basis of 85% lower limit of the laboratory normal range.

Kidney biopsy specimens were examined in the pathology departments of the participating hospitals. The degree of disease activity and chronicity was analyzed according to the previously published C3G histologic index.^4,12^

This study was approved by the Institutional Review Board of Hospital Universitario 12 de Octubre (CEI16/266) and was conducted in accordance with the Declaration of Helsinki. Written informed consent was obtained from participants or their parents to participate in the study.

### Definitions and Outcome

Baseline was defined as the time at which the diagnosis of C3G was made and follow-up period as the interval between kidney biopsy and last outpatient visit or kidney failure.

Nephrotic syndrome was defined as a proteinuria of >3.5 g/d along with serum albumin <3 g/dl. Nephritic syndrome was the combination of hematuria, non-nephrotic proteinuria, hypertension, and kidney function impairment. Asymptomatic urinary abnormalities were defined by the presence of non-nephrotic proteinuria and/or persistent microscopic hematuria >5 erythrocytes per high power field.

The main outcome was kidney failure, defined as an eGFR <15 ml/min per 1.73 m^2^, the need for dialysis or kidney transplantation. Secondary outcomes included complete remission, as an eGFR >60 ml/min per 1.73 m^2^ and proteinuria <0.5 g/24 hours, and partial remission, as a proteinuria reduction >50% plus stabilization/improvement in kidney function. Relapse was defined as the return of pretreatment proteinuria and/or declining kidney function after any remission.

Since we previously found that baseline eGFR, proteinuria, and histologic total chronicity score were the main predictors of kidney failure,^19^ the study population was divided into different clinical profiles by combining these predictors: eGFR under/above 30, proteinuria under/above 3.5 g/d, and total chronicity score under/above 4.

### Statistical Analyses

This was a retrospective, longitudinal, multicenter, observational cohort study. Descriptive statistics are presented as mean±SD or median and interquartile ranges (IQR) for continuous variables and frequencies or percentages for categorical variables. Comparisons of continuous variables between two groups were assessed by using the unpaired *t* test or the Mann–Whitney *U* test, where appropriate. Chi-squared test or Fisher exact test was used for categorical variables.

The incidence rate was calculated as average annual incidence over the study period. The numerator included all patients diagnosed with C3G during each distinct year of the study period. The denominator was calculated based on an estimate considering the number of hospitals participating in the study and the population covered by these hospitals, assuming that all patients during the specified year were followed up for the entire year without loss to follow-up.

For the evaluation of the change in eGFR and proteinuria over time, a subgroup of 85 patients who had at least four consecutive measurements of eGFR and proteinuria was used. Estimation of eGFR slope and change in proteinuria over time were analyzed with linear mixed-effects models. Negative or positive values of these parameters indicated an increase/decrease over time.

Subject-specific longitudinal trajectory plots and locally weighted smoothing plots were used to represent the change of eGFR and proteinuria over follow-up.

Distributions of time to kidney failure were depicted by survival curves using the Kaplan–Meier method, and the survival curves by clinical profiles were compared using the log-rank test.

A *P* value <0.05 was considered to be significant. Analyses were performed using IBM SPSS Statistics 24.0 and R software v.3.6.3.

## Results

### Estimated Incidence and Cohort Description

The total study group consisted of 115 patients after excluding patients not fulfilling diagnostic criteria for C3G (*n*=21), those without alternative complement pathway genetic studies (*n*=20), and those with underlying monoclonal gammopathy (*n*=23).

Assuming an estimated referral population of 7 million over the 25-year study period in the participating hospitals, this equated to an annual incidence of biopsy-proven C3G of almost 1 case/million per year.

The median age was 30 years (IQR 19–50), and 51 subjects (44%) were female (Supplemental Table 1). Twenty-eight (24%) were pediatric patients. Forty-one (36%) patients had a baseline eGFR<30, and 47 (41%) patients had baseline proteinuria ≥3.5 g/d. Nephrotic syndrome was the most common clinical presentation. Regarding the histologic characteristics, 46 (40%) patients had a total activity score ≥9, and 44 (38%) patients had a total chronicity score ≥4. Supplemental Table 2 presents the pathogenic variants in complement genes and acquired abnormalities in the study population.

**Figure 1:**
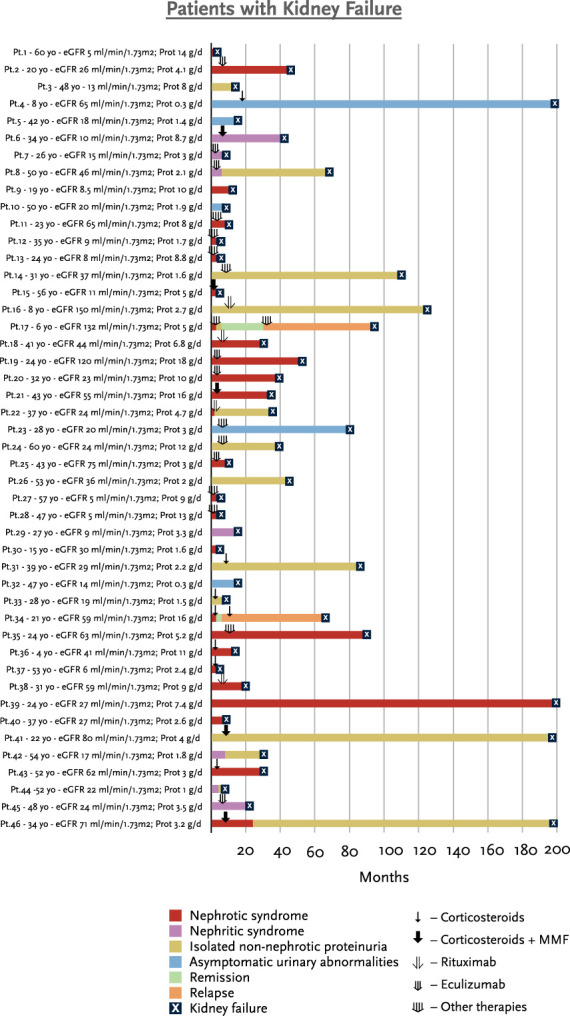
**Detailed description of patients who reached kidney failure at the last follow-up in the overall cohort (*n*=46, out of total 115 patients).** Each Pt is identified by a correlative number (*e.g.*, Pt.1, Pt.2...). Age at diagnosis is shown (yo: years old). eGFR at baseline is expressed as ml/min per 1.73 m^2^. Prot at baseline is expressed as grams/day (g/d). Each bar represents the clinical course of each patient, and the colors represent the type of clinical presentation/outcome over time. Each arrow represents the type of immunosuppressive therapy prescribed and the time at which the treatment was first prescribed. Prot, Proteinuria; Pt, patient.

**Figure 2:**
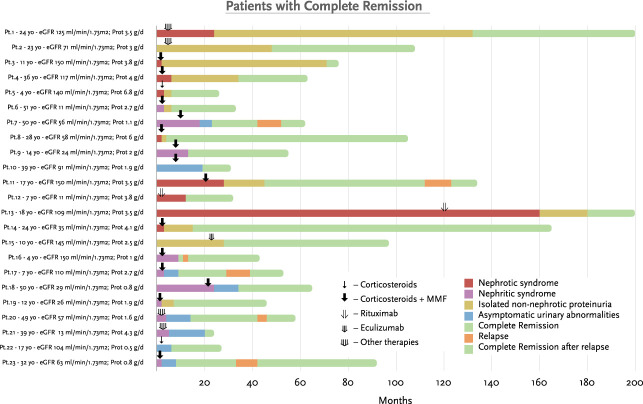
**Detailed description of patients who achieved complete remission at the last follow-up in the overall cohort (*n*=46, out of total 115 patients).** Each Pt is identified by a correlative number (*e.g.*, Pt.1, Pt.2...). Age at diagnosis is shown (yo: years old). eGFR at baseline is expressed as ml/min per 1.73 m^2^. Prot at baseline is expressed as grams/day (g/d). Each bar represents the clinical course of each patient, and the colors represent the type of clinical presentation/outcome over time. Each arrow represents the type of immunosuppressive therapy prescribed and the time at which the treatment was first prescribed. Prot, Proteinuria; Pt, patient.

Over a median follow-up of 49 months (IQR 24–112), 23 (20%) patients achieved complete remission, 31 (27%) patients achieved partial remission, and 46 (40%) patients reached kidney failure. Figure 1 shows the individual clinical course of patients who reached kidney failure at the last follow-up. Most of these patients had significant kidney function impairment at baseline, together with heavy proteinuria, reaching kidney failure before 40 months. Two (4%) patients reached kidney failure after developing a clinical relapse. Figure 2 shows the individual clinical course of patients who achieved complete remission at the last follow-up. The degree of proteinuria decreased during follow-up, and of note, six of the 23 (26%) patients had a clinical relapse during the tapering phase of immunosuppressive treatment.

### Characteristics According to Baseline Clinical Profiles

On the basis of eGFR, proteinuria, and the total disease chronicity in kidney biopsy, the study population was divided into eight clinical profiles.

**Figure 3:**
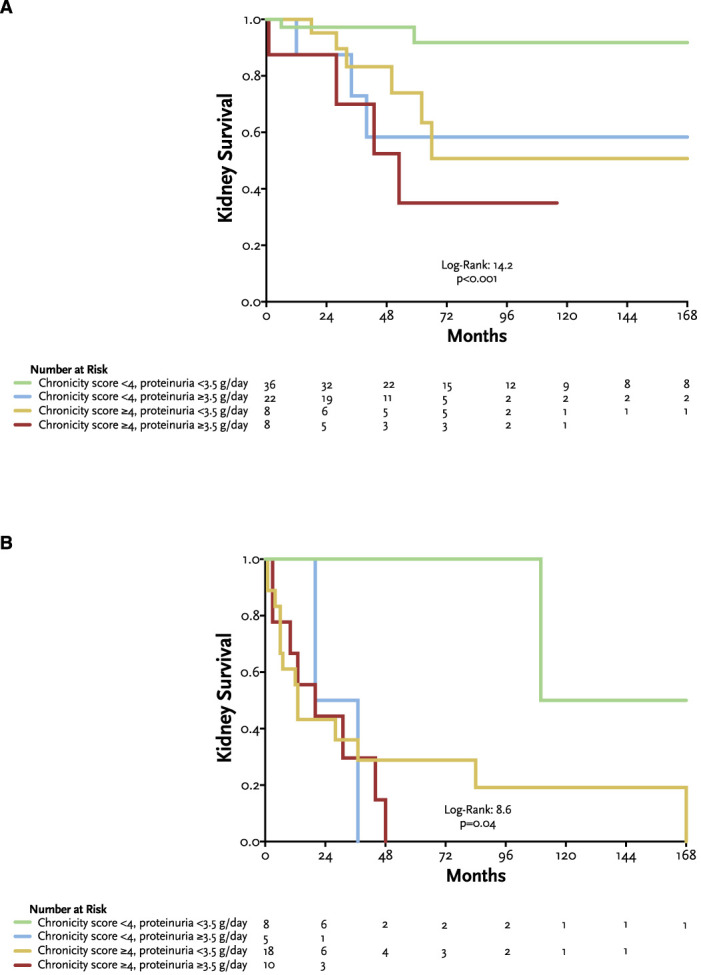
**Kidney survival according to clinical profiles.** (A) Kaplan–Meier curves for kidney survival in patients with baseline eGFR ≥30 ml/min per 1.73 m^2^, according to clinical profiles. (B) Kaplan–Meier curves for kidney survival in patients with baseline eGFR <30 ml/min per 1.73 m^2^, according to clinical profiles.

Of the 74 (64%) patients with baseline eGFR ≥30: 3 (48%) had a chronicity score <4 and proteinuria <3.5g/day, 22 (30%) had a chronicity score <4 and proteinuria ≥3.5 g/d, 8 (11%) had a chronicity score ≥4 and proteinuria <3.5 g/d, and 8 (11%) had a chronicity score ≥4 and proteinuria ≥3.5 g/d (Table 1). As expected, significant differences were observed in the clinical presentation across groups, the frequency of hypoalbuminemia and proteinuria. In addition, differences were also observed in the total disease activity being lower in patients with a chronicity score <4 and proteinuria <3.5 g/d. Figure 3A shows the survival curves of patients with baseline eGFR ≥30, according to clinical profiles. Kidney survival was significantly higher in patients with a chronicity score <4 and proteinuria <3.5 g/d.

**Table 1:** Clinical characteristics of patients with baseline eGFR ≥30 ml/min per 1.73 m^2^, according to clinical profiles at baseline

Characteristic	Chronicity Score <4 and Proteinuria <3.5 g/d (*n*=36)	Chronicity score <4 and proteinuria ≥3.5 g/d (*n*=22)	Chronicity score ≥4 and proteinuria <3.5 g/d (*n*=8)	Chronicity score ≥4 and proteinuria ≥3.5 g/d (*n*=8)	*P*
**Baseline**					
Age, yr					0.31
*<18*	16 (44)	5 (23)	2 (25)	2 (25)	
*≥18*	20 (56)	17 (77)	6 (75)	6 (75)	
Sex, *N* (%)					0.86
*Female*	17 (47)	12 (55)	4 (50)	5 (63)	
*Male*	19 (53)	10 (45)	4 (50)	3 (38)	
Hypertension, *N* (%)					0.49
*Yes*	16 (44)	14 (64)	4 (50)	5 (63)	
*No*	20 (56)	8 (36)	4 (50)	3 (38)	
Clinical presentation, *N* (%)					<0.001
*Nephrotic syndrome*	0 (0)	17 (77)	0 (0)	6 (74)	
*Nephritic syndrome*	9 (25)	3 (13)	0 (0)	1 (13)	
*Isolated non-nephrotic proteinuria*	16 (44)	1 (5)	4 (50)	1 (13)	
*Asymptomatic urinary abnormalities*	11 (31)	1 (5)	4 (50)	0 (0)	
Serum albumin (g/dl), *N* (%)					<0.001
*<3.5*	15 (42)	21 (95)	3 (38)	7 (88)	
*≥3.5*	21 (58)	1 (5)	5 (62)	1 (12)	
Serum C3 (mg/dl), *N* (%)					0.91
*<77*	24 (67)	16 (73)	5 (63)	6 (75)	
*≥77*	12 (33)	6 (27)	3 (37)	2 (25)	
Proteinuria (g/24 h), *N* (%)					<0.001
*<3.5*	36 (100)	0 (0)	8 (100)	0 (0)	
*≥3.5*	0 (0)	22 (100)	0 (0)	8 (100)	
Microscopic hematuria, *N* (%)					0.74
*Yes*	28 (78)	18 (82)	6 (75)	5 (63)	
*No*	8 (22)	4 (18)	2 (25)	3 (38)	
**Alternative complement pathway studies**					
Complement pathogenic variants, *N* (%)	8 (22)	2 (9)	3 (38)	3 (38)	0.22
Variants of unknown significance, *N* (%)	14 (39)	10 (45)	0 (0)	2 (25)	0.11
Antibodies against complement components, *N* (%)	10 (28)	8 (36)	2 (25)	2 (25)	0.87
**Kidney biopsy**					
Histologic subtype, *N* (%)					0.18
*C3GN*	29 (81)	19 (86)	6 (75)	4 (50)	
*DDD*	7 (19)	3 (14)	2 (25)	4 (50)	
C3G histologic index—total activity score					0.01
*<9*	29 (81)	12 (55)	2 (25)	4 (50)	
*≥9*	7 (19)	10 (45)	6 (75)	4 (50)	
C3G histologic index—total chronicity score					<0.001
*<4*	36 (100)	22 (100)	0 (0)	0 (0)	
*≥4*	0 (0)	0 (0)	8 (100)	8 (100)	
**Treatment**					
RAS blockade	33 (92)	20 (91)	6 (75)	6 (75)	0.37
*ACEI*	21 (58)	12 (54)	4 (50)	4 (50)	
*ARB*	5 (14)	4 (18)	1 (12)	2 (25)	
*Both*	7 (19)	4 (18)	1 (12)	0 (0)	
Nonimmunosuppressive therapy	3 (8)	1 (4)	1 (12)	0 (0)	0.73
Corticosteroids only	6 (17)	2 (9)	2 (25)	1 (12)	0.72
Corticosteroids plus MMF	15 (42)	13 (59)	4 (50)	4 (50)	0.64
Rituximab	3 (8)	2 (9)	0 (0)	0 (0)	0.68
Anti-C5	2 (6)	2 (9)	1 (12)	0 (0)	0.74
Other immunosuppressive therapy	7 (19)	2 (9)	0 (0)	3 (37)	0.15
**Outcomes at the last follow-up**					
Follow-up, mo	57 (37–129)	46 (30–68)	36 (15–101)	80 (23–162)	0.32
Complete remission	10 (28)	6 (27)	1 (12)	0 (0)	0.14
Partial remission	14 (39)	6 (27)	3 (38)	2 (25)	0.76
Kidney failure	5 (14)	6 (27)	4 (50)	4 (50)	0.04

C3GN, C3 glomerulonephritis; DDD, dense deposit disease; C3G, C3 glomerulopathy; RAS, renin-angiotensin system; ACEI, angiotensin-converting enzyme inhibitor; ARB, angiotensin receptor blocker; MMF, mycophenolate mofetil.

Of the 41 (36%) patients with baseline eGFR <30: 8 (20%) had a chronicity score <4 and proteinuria <3.5 g/d, 5 (12%) had a chronicity score <4 and proteinuria ≥3.5 g/d, 18 (44%) had a chronicity score ≥4 and proteinuria <3.5 g/d, and 10 (24%) had a chronicity score ≥4 and proteinuria ≥3.5 g/d (Table 2). Most of these patients were adults, and the presence of alternative complement pathway pathogenic variants was significantly lower in patients with a chronicity score <4 and proteinuria <3.5 g/d. This latter group was further characterized by a trend toward a lower frequency in the prescription of renin-angiotensin system blockade and a higher frequency in the prescription of corticosteroids plus mycophenolate mofetil (MMF) as the immunosuppressive regimen. The probability of complete remission was significantly higher in patients with lower chronicity and proteinuria. Figure 3B shows the survival curves of patients with baseline eGFR <30, according to clinical profiles. Similarly, kidney survival was significantly higher in patients with a chronicity score <4 and proteinuria <3.5 g/d.

**Table 2:** Clinical characteristics of patients with baseline eGFR <30 ml/min per 1.73 m^2^, according to clinical profiles at baseline

Characteristic	Chronicity score <4 and proteinuria <3.5 g/d (*n*=8)	Chronicity score <4 and proteinuria ≥3.5 g/d (*n*=5)	Chronicity score ≥4 and proteinuria <3.5 g/d (*n*=18)	Chronicity score ≥4 and proteinuria ≥3.5 g/d (*n*=10)	*P*
**Baseline**					
Age, yr					0.004
*<18*	3 (38)	0 (0)	0 (0)	0 (0)	
*≥18*	5 (62)	5 (100)	18 (100)	10 (100)	
Sex, *N* (%)					0.95
*Female*	2 (25)	2 (40)	6 (33)	3 (30)	
*Male*	6 (75)	3 (60)	12 (67)	7 (70)	
Hypertension, *N* (%)					0.10
*Yes*	5 (63)	5 (100)	17 (94)	9 (90)	
*No*	3 (37)	0 (0)	1 (6)	1 (10)	
Clinical presentation, *N* (%)					0.02
*Nephrotic syndrome*	0 (0)	1 (20)	0 (0)	6 (60)	
*Nephritic syndrome*	5 (63)	4 (80)	8 (45)	4 (40)	
*Isolated non-nephrotic proteinuria*	2 (25)	0 (0)	6 (33)	0 (0)	
*Asymptomatic urinary abnormalities*	1 (12)	0 (0)	4 (22)	0 (0)	
Serum albumin (g/dl), *N* (%)					0.06
*<3.5*	7 (88)	5 (100)	10 (56)	9 (90)	
*≥3.5*	1 (12)	0 (0)	8 (44)	1 (10)	
Serum C3 (mg/dl), *N* (%)					0.13
*<77*	6 (75)	4 (80)	8 (44)	3 (30)	
*≥77*	2 (25)	1 (20)	10 (56)	7 (70)	
Proteinuria (g/24 h), *N* (%)					<0.001
*<3.5*	8 (100)	0 (0)	18 (100)	0 (0)	
*≥3.5*	0 (0)	5 (100)	0 (0)	10 (100)	
Microscopic hematuria, *N* (%)					0.52
*Yes*	7 (88)	5 (100)	13 (72)	8 (80)	
*No*	1 (12)	0 (0)	5 (28)	2 (20)	
**Alternative complement pathway studies**					
Complement pathogenic variants, *N* (%)	0 (0)	3 (60)	3 (17)	1 (10)	0.04
Variants of unknown significance, *N* (%)	2 (25)	2 (40)	8 (44)	3 (30)	0.76
Antibodies against complement components, *N* (%)	2 (25)	2 (40)	6 (33)	1 (10)	0.51
**Kidney biopsy**					
Histologic subtype, *N* (%)					0.68
*C3GN*	8 (100)	4 (80)	16 (89)	9 (90)	
*DDD*	0 (0)	1 (20)	2 (11)	1 (10)	
C3G histologic index—total activity score					0.83
*<9*	4 (50)	2 (40)	11 (61)	5 (50)	
*≥9*	4 (50)	3 (60)	7 (39)	5 (50)	
C3G histologic index—total chronicity score					<0.001
*<4*	8 (100)	5 (100)	0 (0)	0 (0)	
*≥4*	0 (0)	0 (0)	18 (100)	10 (100)	
**Treatment**					
RAS blockade	4 (50)	5 (100)	16 (89)	8 (80)	0.08
*ACEI*	3 (38)	4 (80)	15 (83)	4 (40)	
*ARB*	1 (12)	1 (20)	1 (6)	2 (20)	
*Both*	0 (0)	0 (0)	0 (0)	2 (20)	
Nonimmunosuppressive therapy	0 (0)	0 (0)	9 (50)	4 (40)	0.03
Corticosteroids only	0 (0)	1 (20)	3 (17)	0 (0)	0.32
Corticosteroids plus MMF	5 (63)	1 (20)	2 (11)	2 (20)	0.04
Rituximab	1 (12)	0 (0)	0 (0)	1 (10)	0.43
Anti-C5	0 (0)	1 (20)	1 (11)	2 (20)	0.57
Other immunosuppressive therapy	2 (25)	2 (40)	2 (11)	1 (10)	0.39
**Outcomes at the last follow-up**					
Follow-up, mo	36 (18–47)	6 (3–27)	12 (6–27)	20 (5–43)	0.12
Complete remission	5 (63)	0 (0)	1 (6)	0 (0)	<0.001
Partial remission	2 (25)	1 (20)	2 (11)	1 (10)	0.76
Kidney failure	1 (12)	3 (60)	14 (78)	9 (90)	0.003

C3GN, C3 glomerulonephritis; DDD, dense deposit disease; C3G, C3 glomerulopathy; RAS, renin-angiotensin system; ACEI, angiotensin-converting enzyme inhibitor; ARB, angiotensin receptor blocker; MMF, mycophenolate mofetil.

Initial doses of each immunosuppressive regimen, duration, and adverse events, according to baseline eGFR, are presented in Supplemental Table 3. No significant differences were observed in the median initial doses received across groups.

Regarding the treatment-related adverse events, infectious complications were the most common (17%)—particularly urinary tract infections (10%) followed by cardiovascular events (13%), cytopenia (10%), new-onset diabetes mellitus (4%), and avascular necrosis of the hip (4%).

### Change in eGFR and Proteinuria Over Time

For the evaluation of the slope of eGFR and change in proteinuria over follow-up time, a subgroup of 85 patients with similar characteristics to those of the overall cohort was used (Supplemental Table 1).

Nineteen (22%) patients achieved complete remission, 29 (34%) patients achieved partial remission, and 25 (29%) patients reached kidney failure. The median eGFR slope of this latter group was -6.5 ml/min per 1.73 m^2^ per year (IQR −1.6 to −17). Supplemental Figure 1 shows the individual-specific longitudinal trajectories of eGFR for each treatment category, according to the development of kidney failure. A progressive increase in eGFR was observed in the group of patients not reaching kidney failure. This rise in eGFR was steeper in patients treated with corticosteroids plus MMF, whereas in the rest of therapeutic groups, the eGFR exhibited a trend toward a nonlinear quadratic pattern.

On the other hand, the median change in proteinuria of patients reaching kidney failure was 2.5 g/d per year (IQR 0.5–4.5). Supplemental Figure 2 shows the individual-specific longitudinal trajectories of proteinuria for each treatment category, according to the development of kidney failure. Patients who showed a progressive reduction in proteinuria over time did not reach kidney failure.

A trend toward a decrease in C3 levels was also observed in patients who reached kidney failure (Supplemental Figure 3). In addition, when kidney survival was analyzed according to the categories of serum C3 levels at the end of follow-up, a nonsignificant trend was observed toward a better survival in patients with C3 levels ≥77 mg/dl (Supplemental Figure 4).

### Patterns of eGFR Decline

On the basis of the slope of eGFR over time, patients were classified as those with a faster eGFR decline (≥5 ml/min per 1.73 m^2^ per year), those with a slower eGFR decline (<5 ml/min per 1.73 m^2^ per year), and those without decline (*i.e.* positive slope in eGFR over follow-up) (Table 3).

**Table 3:** Clinical characteristics of patients according to the rate of decline of eGFR, in a subgroup of 85 patients with consecutive measurements of eGFR and proteinuria over time

Characteristic	Faster Decline (eGFR Slope ≥5 ml/min per 1.73 m^2^ per year) (*n*=26)	Slower Decline (eGFR Slope <5 ml/min per 1.73 m^2^ per year) (*n*=23)	No Progression (*n*=36)	*P*
**Baseline**				
Age, yr				0.81
*<18*	9 (35)	6 (26)	11 (31)	
*≥18*	17 (65)	17 (74)	25 (69)	
Sex, *N* (%)				0.005
*Female*	18 (69)	11 (48)	10 (28)	
*Male*	8 (31)	12 (52)	26 (72)	
Hypertension, *N* (%)				0.79
*Yes*	15 (58)	12 (52)	22 (61)	
*No*	11 (42)	11 (48)	14 (39)	
Clinical presentation, *N* (%)				0.24
*Nephrotic syndrome*	14 (54)	10 (44)	10 (28)	
*Nephritic syndrome*	5 (19)	5 (22)	15 (42)	
*Isolated non-nephrotic proteinuria*	4 (15)	2 (9)	5 (14)	
*Asymptomatic urinary abnormalities*	3 (12)	6 (26)	6 (17)	
eGFR at diagnosis, ml/min per 1.73 m^2^				0.25
*<30*	6 (23)	5 (22)	14 (39)	
*≥30*	20 (77)	18 (78)	22 (61)	
Serum albumin, g/dl				0.54
*<3.5*	18 (69)	13 (57)	25 (69)	
*≥3.5*	8 (31)	10 (43)	11 (31)	
Serum C3, mg/dl				0.47
*<77*	19 (73)	13 (57)	24 (67)	
*≥77*	7 (27)	10 (43)	12 (33)	
Proteinuria, g/24 h				0.87
*<3.5*	14 (54)	14 (61)	21 (58)	
*≥3.5*	12 (46)	9 (39)	15 (42)	
Microscopic hematuria, *N* (%)				0.12
*Yes*	17 (65)	19 (63)	31 (86)	
*No*	9 (35)	4 (17)	5 (14)	
**Alternative complement pathway studies**				
Complement pathogenic variants, *N* (%)	3 (12)	9 (39)	4 (11)	0.01
Variants of unknown significance, *N* (%)	13 (50)	6 (26)	17 (47)	0.17
Antibodies against complement components, *N* (%)	11 (42)	4 (17)	9 (25)	0.13
**Kidney biopsy**				
Histologic subtype				0.27
*C3GN*	19 (73)	19 (83)	32 (89)	
*DDD*	7 (27)	4 (17)	4 (11)	
C3G histologic index—total activity score				0.43
*<9*	16 (62)	13 (57)	26 (72)	
*≥9*	10 (38)	10 (43)	10 (28)	
C3G histologic index—total chronicity score				0.29
*<4*	16 (62)	13 (57)	27 (75)	
*≥4*	10 (38)	10 (43)	9 (25)	
**Treatment**				
RAS blockade	22 (85)	21 (91)	31 (86)	0.76
Nonimmunosuppressive therapy	4 (15)	5 (22)	3 (8)	0.34
Corticosteroids only	5 (19)	1 (4)	6 (17)	0.27
Corticosteroids plus MMF	8 (31)	9 (39)	23 (64)	0.02
Rituximab	3 (12)	2 (9)	1 (3)	0.38
Anti-C5	3 (12)	2 (9)	2 (6)	0.69
Other immunosuppressive therapy	3 (12)	4 (17)	1 (3)	0.15
**Outcomes**				
Follow-up, mo	35 (15–64)	73 (37–186)	38 (17–51)	0.01
Kidney failure	16 (62)	9 (39)	0 (0)	<0.001

C3GN, C3 glomerulonephritis; DDD, dense deposit disease; C3G, C3 glomerulopathy; RAS, renin-angiotensin system; MMF, mycophenolate mofetil.

**Figure 4:**
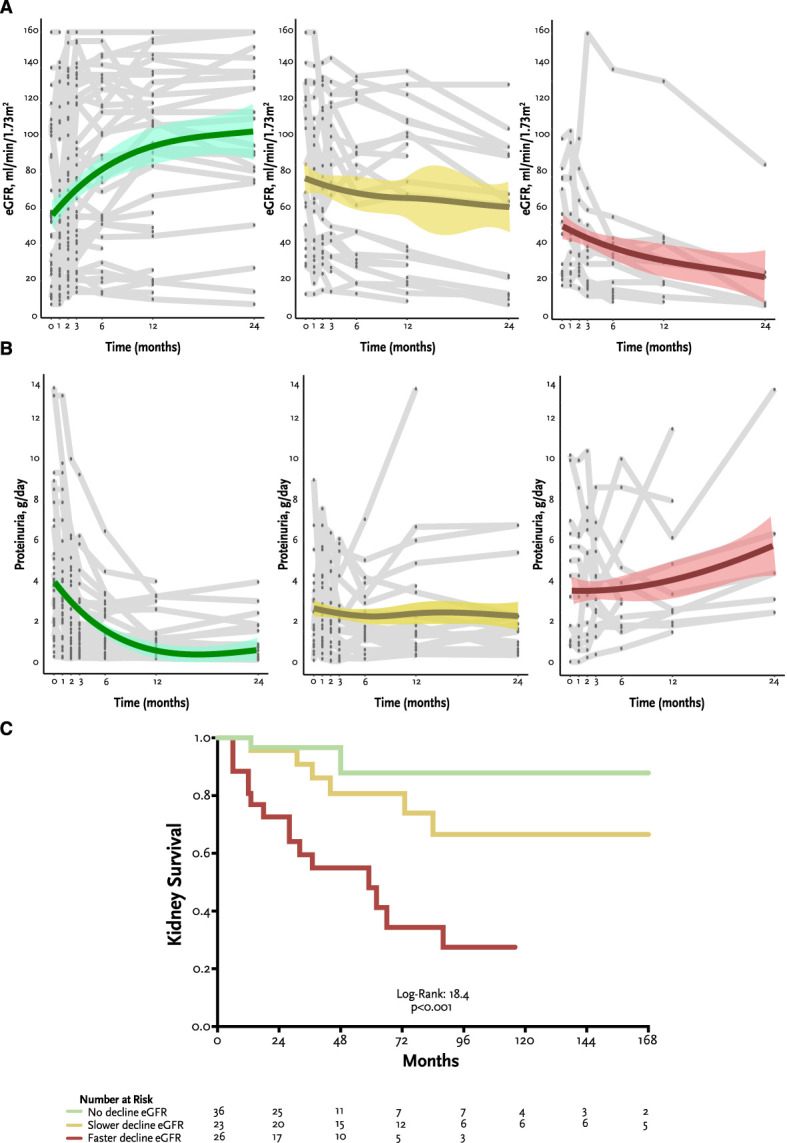
**Patterns of eGFR and proteinura change over time.** (A) Subject-specific longitudinal trajectories of eGFR over follow-up (time points are 0, 1, 2, 3, 6, 12, and 24 months) in a subgroup of 85 patients with consecutive measurements of eGFR and proteinuria over time. The gray lines show individual subject trajectories, and the color lines show LOESS with the corresponding 95% confidence interval. The first graph (in green) represents patients with no decline of eGFR over time; the second graph (in yellow) represents patients with a slower eGFR decline (<5 ml/min per 1.73 m^2^ per year); and the third graph represents patients with a faster eGFR decline (≥5 ml/min per 1.73 m^2^ per year); (B) Subject-specific longitudinal trajectories of 24-hour proteinuria over follow-up (time points are 0, 1, 2, 3, 6, 12, and 24 months) in a subgroup of 85 patients with consecutive measurements of eGFR and proteinuria over time. The gray lines show individual subject trajectories, and the color lines show LOESS with the corresponding 95% confidence interval. The first graph (in green) represents patients with no decline of eGFR over time; the second graph (in yellow) represents patients with a slower eGFR decline (<5 ml/min per 1.73 m^2^ per year); the third graph represents patients with a faster eGFR decline (≥5 ml/min per 1.73 m^2^ per year). (C) Kaplan–Meier curves for kidney survival according to the rate of decline of eGFR (patients with faster eGFR decline, slower eGFR decline, and no eGFR decline). LOESS, locally weighted smoothing plots.

Patients with a faster eGFR decline were predominantly women with dense deposit disease, presenting more frequently with nephrotic syndrome, and were less frequently treated with corticosteroids plus MMF. These patients exhibited a progressive increase in proteinuria as eGFR declined over time (Figure 4, A and B). A faster rate of decline in eGFR was associated with higher probability of kidney failure (Table 3). Figure 4C shows the survival curves according to the rate of decline of eGFR. Kidney survival was significantly lower in patients with a faster decline of eGFR.

## Discussion

In this study, we described the spectrum of clinical profiles and patterns of progression of kidney disease in a large cohort of patients with C3G. Our study confirms an estimated incidence rate of C3G of almost one case per million population per year. Second, we showed the individual clinical evolution of patients who achieved complete remission and the temporal relationship with each treatment initiation. Third, we analyzed the different clinical profiles of patients at the time of clinical diagnosis by combining the main predictors of outcomes (eGFR, proteinuria, and disease chronicity in kidney biopsy). Kidney survival was significantly higher in patients with a chronicity score <4 and proteinuria <3.5 g/d, regardless of eGFR at presentation. Finally, we assessed the different patterns of eGFR change over time, and as expected, a faster rate of decline in eGFR was associated with higher probability of reaching kidney failure.

Eight clinical profiles of patients were identified. Among the group of patients with a baseline eGFR ≥30, those with lower disease chronicity in kidney biopsy were more likely to respond to immunosuppressive regimens and to achieve remission (partial/complete). Most of these patients were treated with corticosteroids plus MMF. Although the mechanism of action of this therapeutic regimen is not well understood, some studies have found beneficial effects with this regimen,^18,20^ while others have not.^7,9^ Kidney failure was less likely in patients with lower disease chronicity in kidney biopsy and lower proteinuria. These findings are in line with others^9,10,21^ and suggest that the importance of proteinuria in C3G not only as a marker of disease severity but also as a predictor of kidney damage.^11,22,23^ On the other hand, the percentage of patients who reached kidney failure in the group of patients with a baseline eGFR ≥30 was higher in those with concomitant higher disease chronicity in kidney biopsy, which points toward a potential point of no return in the disease.^12^

The group of patients with a baseline eGFR <30 was predominantly comprised by adults. Interestingly, within this group of patients with lower disease chronicity and proteinuria, five (63%) patients achieved complete remission at the last follow-up, pointing toward a reversible kidney damage at the time of clinical diagnosis. Similarly, these patients were mostly treated with corticosteroids plus MMF. As expected, the probability of kidney failure was higher in those with higher disease chronicity and proteinuria. We speculate that this could be due to a more aggressive clinical phenotype or a late diagnosis of the disease.

Another interesting observation in our study was that a subset of patients had a clinical relapse of the disease after having achieved some degree of remission with immunosuppressive therapy. Although the overall clinical course is slowly progressive in most of the patients, disease exacerbations may also occur in the context of superimposed alternative complement activation by intercurrent factors, such as infections.^16^ Thus, patients with C3G require a close follow-up after diagnosis, although the most appropriate strategy or biomarker to monitor relapses of the disease remains to be defined.

Finally, we analyzed the clinical patterns of eGFR change over time and confirmed the inverse relationship between eGFR change and proteinuria among different subgroups of patients with C3G on the basis of the rate of decline.^11^ Three different groups were distinguished: faster, lower, and no decline of eGFR over time. A large proportion of patients with a faster decline corresponded to dense deposit disease cases—although the differences did not reach statistical significance—and had been treated with an immunosuppressive regimen different than corticosteroids plus MMF. Patients without progression of kidney disease were mostly men, with nephritic syndrome as the most frequent presentation and low degree of chronicity in kidney biopsy.

According to our results, the decline in eGFR over time is not uniformly linear in patients with C3G, and the rate of decline can vary widely between individuals. Therefore, these different patterns of progression should be considered for both clinical studies and trial recruitment. Moreover, from a clinical standpoint, they could also help to titrate the intensity of treatments or to schedule the initiation of dialysis or kidney transplantation, particularly at later stages of the disease.^24^

Taken together, our study suggests that the clinical profile of patients who could benefit most from anticomplement therapies would be those with lower degree of disease chronicity in kidney biopsy. In a subset of patients with a low eGFR at clinical presentation, the kidney function may recover—at least partially—and thus, in our opinion this sole criterion should not be used to decide whether to treat a patient with C3G with future anticomplement drugs. One might speculate that once these new anticomplement agents were prescribed, those who experienced a decrease in proteinuria or a change in the rate of decline of eGFR would be those associated with a better renal prognosis in the medium and long term, although further studies are warranted to better address this issue.

This study is subject to limitations. First, owing to the observational and retrospective nature of the study, no causal relationships can be established. Second, given the retrospective nature of this study and the large period covered, some cases of C3G might not have been correctly identified during follow-up in the participating centers, which could have affected the estimated incidence rate. Third, although in this study, we were able to identify patients with C3G with poor short-term and medium-term kidney prognosis, longer follow-up may be necessary to fully capture the natural history of the disease in some patients. Despite these limitations, this study further contributes to the understanding of the disease by providing additional information for predicting outcomes in clinical settings.

In conclusion, kidney survival was significantly higher in patients with a disease chronicity score <4 and proteinuria <3.5 g/d, regardless of baseline eGFR; a faster eGFR decline was associated with higher probability of kidney failure; and patients with a progressive reduction in proteinuria over time did not reach kidney failure. Nevertheless, several questions remain open for further research, and therefore, prospective studies are warranted to better elucidate them.

## Supplementary Material

SUPPLEMENTARY MATERIAL

## Data Availability

The data underlying this article will be shared on reasonable request to the corresponding author.

## Supplemental Material

Supplemental Methods.

Supplemental Table 1. Clinical characteristics of patients according to study population subgroups.

Supplemental Table 2. Pathogenic variants in complement genes and acquired abnormalities in the study population.

Supplemental Table 3. Initial dose of treatment regimens, duration, and adverse events, according to age groups.

Supplemental Figure 1. Subject-specific longitudinal trajectories of eGFR over follow-up, according to immunosuppressive treatments.

Supplemental Figure 2. Subject-specific longitudinal trajectories of 24 hours proteinuria over follow-up, according to immunosuppressive treatments.

Supplemental Figure 3. Subject-specific longitudinal trajectories of C3 in adult and pediatric patients, according to the development of kidney failure.

Supplemental Figure 4. Kaplan–Meier curves for kidney survival according to serum C3 levels at the last follow-up.
