# Supplementary material for: Clinical Profiles and Patterns of Kidney Disease Progression in C3 Glomerulopathy
Source: Kidney360. 2023 Mar 30;4(5):659–72. doi: 10.34067/KID.0000000000000115 (PMC10278771; doi:10.34067/KID.0000000000000115)
Supplement: SUPPLEMENTARY MATERIAL [file kidney360-4-659-s001.pdf]

Clinical Profiles and Patterns of Kidney Disease Progression in C3 Glomerulopathy

Index to Supplementary Material

1) SUPPLEMENTARY METHODS 2

2) SUPPLEMENTARY TABLES:

Supplementary Table S1: Clinical characteristics of patients according to study population subgroups..3

Supplementary Table S2: Pathogenic variants in complement genes and acquired abnormalities in the study population..4

Supplementary Table S3: Initial dose of treatment regimens, duration and adverse events, according to age groups..5

3) SUPPLEMENTARY FIGURES:

Supplementary Figure S1: Subject-specific longitudinal trajectories of eGFR over follow-up, according to immunosuppressive treatments..6

Supplementary Figure S2: Subject-specific longitudinal trajectories of 24h-proteinuria over follow-up, according to immunosuppressive treatments..7

Supplementary Figure S3: Subject-specific longitudinal trajectories of C3 in adult and pediatric patients, according to the development of kidney failure..................................8

Supplementary Figure S4: Kaplan-Meier curves for kidney survival according to serum C3 levels at last follow-up 9

Supplementary Methods:

Complement genetics and molecular studies

Genomic DNA was prepared from peripheral blood cells according to standard procedures. The entire set of complement genes was analyzed in all samples by next generation sequencing (NGS) using the MiSeq next generation sequencing platform (Illumina, USA). In addition, copy number variation (CNV) analysis of the CFH-CFHR region was performed by Multiplex Ligation-dependent Probe Amplification (MLPA). European non-Finish population from gnomAD database was used as a control population. Variants found in coding and flanking regions were considered; synonym changes were excluded. Variants with a minor allele frequency (MAF) of 1% or lower were identified as rare variants and classified according to their pathogenicity. Only variants in the candidate genes *CFH*, *CFI*, *C3* and *CFB* or genomic rearrangements in the *CFHRs* were considered pathogenic. A variant was categorized as pathogenic if there was experimental evidence of reduced protein levels or altered function, or when at least 4 out of 6 bioinformatic predictors (SIFT, Polyphen, MutTast, MutAss, FATHMM, CADD) indicated pathogenicity. A variant was considered benign when functional data demonstrated normal protein levels and function, or 4 out of 6 predictors classified it as benign. A variant of unknown significance was considered when none of the other two criteria were met.

Serum levels of C3 and C4 were quantified using nephelometric method. The detection of anti-FH antibodies and C3 nephritic factor was performed by ELISA and hemolytic assays.

Supplementary Table S1: Clinical characteristics of patients according to study population subgroups.

	***Overall cohort** (N=115)*	***Subgroup with longitudinal follow-up** (N=85)*
**Baseline**		
Age (years)		
<18	28 (24)	26 (31)
≥18	87 (76)	59 (69)
Sex, N (%)		
Female	51 (44)	39 (46)
Male	64 (56)	46 (54)
Hypertension, N (%)		
Yes	75 (65)	49 (58)
No	40 (35)	36 (42)
Clinical presentation, N (%)		
Nephrotic syndrome	46 (40)	34 (40)
Nephritic syndrome	34 (30)	25 (29)
Isolated non-nephrotic proteinuria	15 (13)	11 (13)
Asymptomatic urinary abnormalities	20 (17)	15 (18)
eGFR at diagnosis (ml/min/1.73m^2), N (%)		
<30	41 (36)	25 (29)
≥30	74 (64)	60 (71)
Serum albumin (g/dl), N (%)		
<3.5	77 (67)	56 (66)
≥3.5	38 (33)	29 (34)
Serum C3 (mg/dl), N (%)		
<77	72 (63)	56 (66)
≥77	43 (37)	29 (34)
Proteinuria (g/24h), N (%)		
<3.5	68 (59)	49 (58)
≥3.5	47 (41)	36 (42)
Microscopic hematuria, N (%)		
Yes	90 (78)	67 (79)
No	25 (22)	18 (21)
**Alternative complement pathway studies**		
Complement pathogenic variants, N (%)	23 (20)	16 (19)
Variants of unknown significance, N (%)	41 (36)	36 (42)
Antibodies against complement components, N (%)	33 (29)	24 (28)
**Kidney biopsy**		
Histologic subtype, N (%)		
C3GN	95 (83)	70 (82)
DDD	20 (17)	15 (18)
C3G Histologic Index - Total Activity score		
<9	69 (60)	55 (65)
≥9	46 (40)	30 (35)
C3G Histologic Index - Total Chronicity score		
<4	71 (62)	56 (66)
≥4	44 (38)	29 (34)
**Treatment**		
RAS blockade	98 (85)	74 (87)
ACEI	67 (58)	53 (62)
ARB	17 (15)	13 (15)
Both	14 (12)	8 (9)
Non-immunosuppressive therapy	18 (16)	12 (14)
Corticosteroids only	15 (13)	12 (14)
Corticosteroids plus MMF	46 (40)	40 (47)
Rituximab	7 (6)	6 (7)
Anti-C5	10 (9)	7 (8)
Other immunosuppressive therapy	19 (17)[a]	8 (9)[b]
**Outcomes at last follow-up**		
Complete remission	23 (20)	19 (22)
Partial remission	31 (27)	29 (34)
Kidney failure	46 (40)	25 (29)

Abbreviations: ACEI: angiotensin converting enzyme inhibitor; ARB: angiotensin receptor blocker; C3G: C3 glomerulopathy; C3GN: C3 glomerulonephritis; DDD: dense deposit disease; eGFR: estimated glomerular filtration rate; MMF: mycophenolate mofetil; RAS: renin-angiotensin system

[a] Including cyclophosphamide-based regimens (n=14), azathioprine (n=2), calcineurin inhibitors (n=3)
[b] Including cyclophosphamide-based regimens (n=5), azathioprine (n=2), calcineurin inhibitors (n=1)

Supplementary Table S2: Summary of pathogenic variants in complement genes and acquired abnormalities in study population

Patient	Genetic variant	Autoantibody against complement component
#3	*C3*: c.1656G>C; p.Trp552Cys (Het)	–
#6	*CFHR1:* Duplic(E2-E6) (Het)	–
#8	–	*C3 nephritic factor*
#12	–	*C3 nephritic factor*
#14	*C3*: c.1379T>G; p.Val460Gly (Het)	–
#15	*CFI*: c.1234G>A; p.Val412Met (Het)	–
#16	–	*C3 nephritic factor*
#18	*C3:* c.1269+1G>A (Het)	–
#20	*C3:* c.2770G>A; p.Gly924Ser (Het)	–
#25	–	*C3 nephritic factor*
#27	*C3:* c.2203C>T; p.Arg735Trp (Het)	–
#28	*CFI:* c.1071T>G; p.Ile357Met (Het)	–
#31	–	*Anti-factor H*
#37	–	*Anti-factor H*
#38	–	*C3 nephritic factor*
#39	*CFH:* c.328G>T; p. Ala110Ser (Hom)	–
#41	–	*C3 nephritic factor*
#42	*CFHR1:* Duplic(PROM-E3) (Het)	–
#45	–	*Anti-factor H*
#47	–	*C3 nephritic factor*
#49	*C3:* c.1898A>G; Lys633Arg (Het)	–
#50		*C3 nephritic factor*
#51	–	*C3 nephritic factor*
#52	–	*Anti-factor H*
#54	*CFHR5:* c.1704T>A; Cys568*	–
#55		*Anti-factor H*
#56	–	*C3 nephritic factor*
#57	–	*C3 nephritic factor*
#58	–	*C3 nephritic factor*
#59	*THBD:* c.127G>A; Ala43Thr (Het)	–
#61	*C3:* c.4339T>C; p.Tyr1447His (Het)	–
#62	–	*C3 nephritic factor*
#63	*CFH:* c.328G>T; p.Ala110Ser (Het)	–
#69	–	*Anti-factor H*
#71	*CFH:* c.1132G>T; p.Gly378* (Het)	–
	ADAMTS13: c.2195C>T; Ala732Val (Het)	–
#72	*THBD:* c.976G>A; p. Val326Met (Het)	–
#73	Hybrid Gene *CFHR3::CFHR1* (Het)	–
#74	–	*C3 nephritic factor*
#75	–	*C3 nephritic factor*
#79	–	*Anti-factor H*
#80	–	*C3 nephritic factor and anti-factor H*
#82	–	*C3 nephritic factor*
#83	–	*C3 nephritic factor*
#84	*C3:* c.C3481A; p.Gln1161Lys (Het)	–
	CFHR5: c.479_480insAA; p.Glu163Kfs*10 (Het)	–
#85	–	*C3 nephritic factor*
#89	–	*C3 nephritic factor*
#90	*CFI:* c.1508_1510del; p.Phe503del (Het)	–
#92	*CFB:* c.724A>C; p.Ile242Leu (Het)	–
#94	–	*C3 nephritic factor*
#95	–	*C3 nephritic factor*
#96	–	*Anti-factor H*
#97	*C3: c.2203C>T;p.Arg735Trp (Het)*	–
#101	–	*C3 nephritic factor*
#102	–	*C3 nephritic factor*
#113	–	*C3 nephritic factor*
#115	*CFHR5: c.486_487dupA; p.Glu63Argfs*35 (Het)*	–

Supplementary Table S3: Initial dose of treatment regimens, duration and adverse events, according to eGFR groups.

	Total (N=115)	*eGFR<30 ml/min/1.73m²* *(N=41)*	*eGFR≥30 ml/min/1.73m²* *(N=74)*	*p*
**Initial dose**				
Corticosteroids, mg/kg/day	0.9 (0.7–1)	0.8 (0.3–1)	1 (1–1.2)	0.13
MMF, g/day	1 (0.75-1.5)	1 (0.75–1.5)	1 (0.75-1.5)	0.81
Rituximab, no. doses	3 (2–4)	2	3 (2–4)	0.19
Cyclophosphamide, mg/kg	8 (1–11)	10 (6–12)	5 (1–11)	0.17
**Duration, months**				
Corticosteroids	9 (3–15)	3 (2–6)	7 (5–15)	0.05
MMF	11 (6–24)	8 (3–15)	15 (7–27)	0.08
Rituximab	3 (2–5)	1	1 (1–2)	0.57
Anti-C5	6 (4–38)	5 (4–24)	13 (2–53)	0.84
Cyclophosphamide	6 (3–10)	2 (1–5)	13 (7–18)	0.006
**Adverse events**				
Infectious complications, N (%)	20 (17)	9 (22)	11 (15)	0.34
Respiratory tract infection	11 (10)	4 (10)	7 (10)	
Meningitis	1 (1)	1 (2)	0 (0)	
Urinary tract infection	3 (3)	1 (2)	2 (3)	
Cytomegalovirus infection	3 (3)	1 (2)	2 (3)	
Herpes Zoster infection	1 (1)	1 (2)	0 (0)	
Abdominal sepsis	1 (1)	1 (2)	0 (0)	
Diabetes mellitus, N (%)	4 (4)	2 (5)	2 (3)	0.52
Cytopenia, N (%)	12 (10)	6 (15)	6 (8)	0.27
Anemia	6 (5)	3 (7)	3 (4)	
Leukopenia	4 (4)	2 (5)	2 (3)	
Thrombocytopenia	2 (2)	1 (2)	1 (1)	
Cardiovascular event, N (%)	15 (13)	7 (17)	8 (11)	0.34
HTN crisis/Malignant HTN	4 (4)	3 (7)	1 (1)	
Arrhythmia	1 (1)	1 (2)	0 (0)	
DVT/PE	3 (3)	0 (0)	3 (4)	
Ischemic cardiomyopathy/ACS	4 (4)	2 (5)	2 (3)	
Acute pulmonary edema	1 (1)	1 (2)	0 (0)	
Stroke	1 (1)	0 (0)	1 (1)	
Valvular heart disease	1 (1)	0 (0)	1 (1)	
Avascular necrosis of hip, N (%)	4 (4)	1 (2)	3 (4)	0.65
Other, N (%)	7 (6)	2 (5)	5 (7)	0.69
Cataracts	1 (1)	0 (0)	1 (1)	
Bone fracture	1 (1)	1 (2)	0 (0)	
Drug infusion reaction/intolerance	1 (1)	0 (0)	1 (1)	
Rhabdomyolysis	1 (1)	0 (0)	1 (1)	
Breast cancer	1 (1)	0 (0)	1 (1)	
Non-melanoma skin cancer	1 (1)	1 (2)	0 (0)	
Suicide attempt	1 (1)	0 (0)	1 (1)	

Abbreviations: ACS: acute coronary syndrome; HTN: hypertension; DVT/TE: deep vein thrombosis / pulmonary thromboembolism;

Supplementary Figure S1: Subject-specific longitudinal trajectories of eGFR over follow-up, according to immunosuppressive treatments.

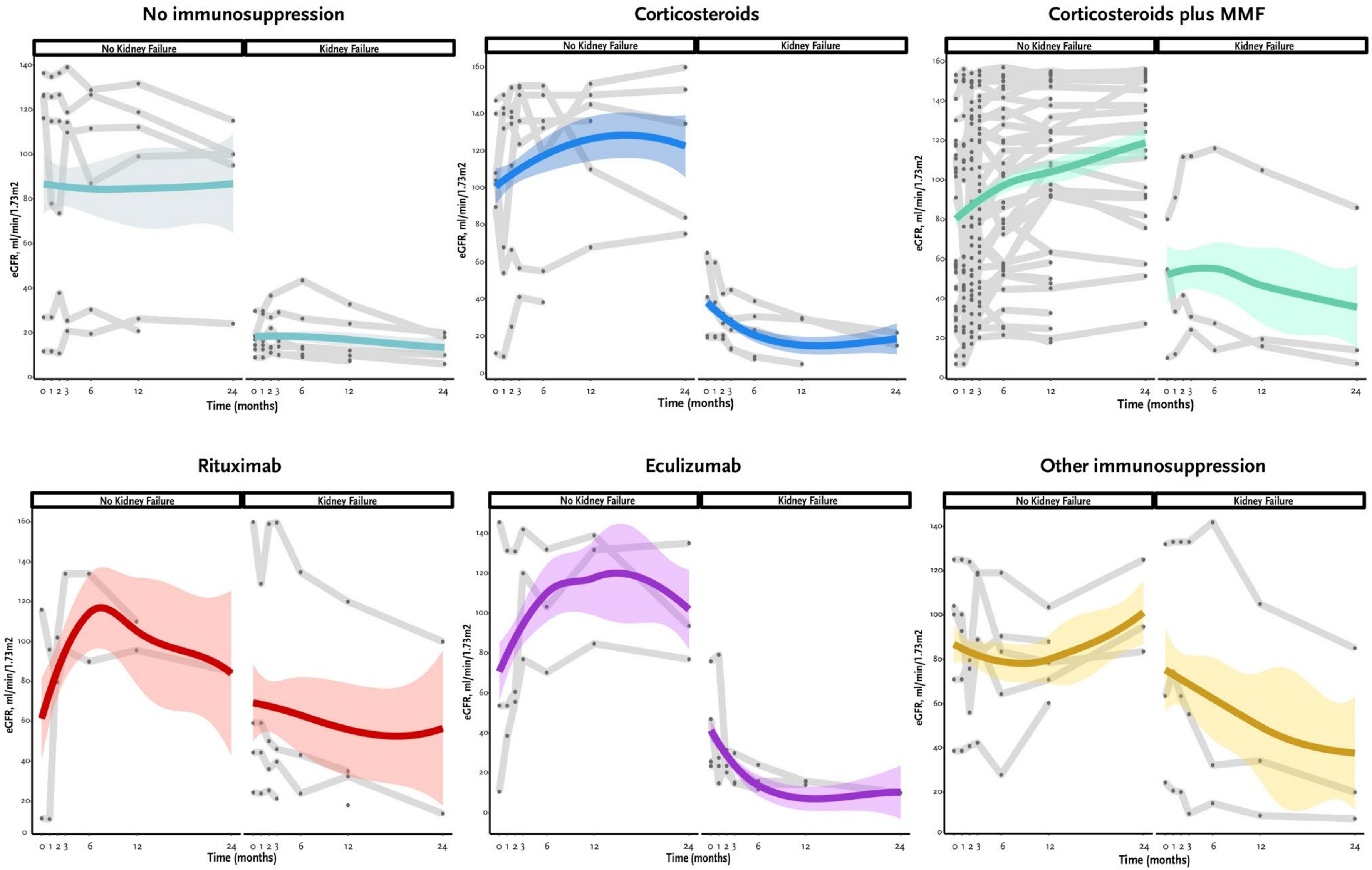

Supplementary Figure S2: Subject-specific longitudinal trajectories of 24h-proteinuria over follow-up, according to immunosuppressive treatments.

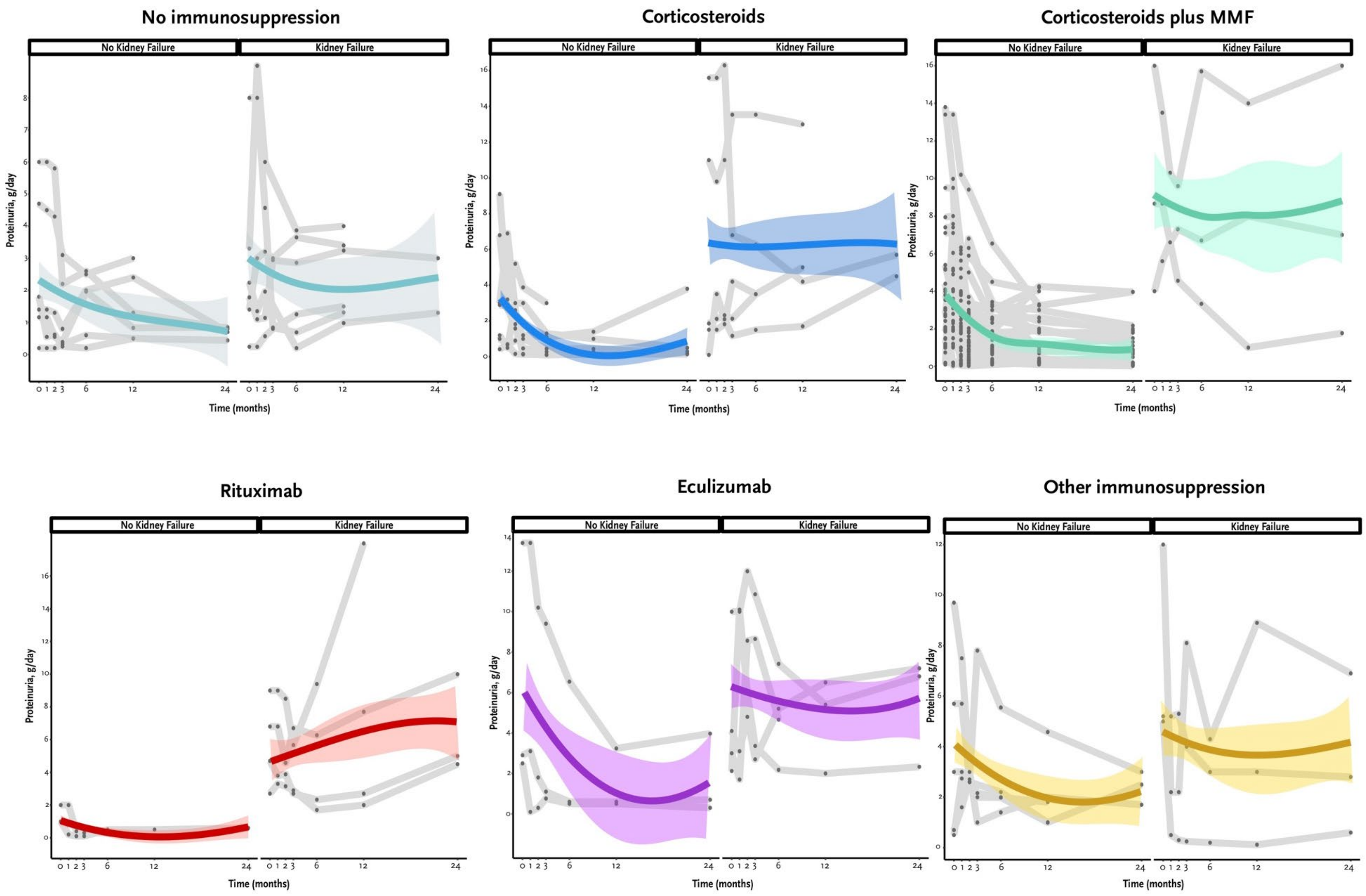

Supplementary Figure S3: A) Subject-specific longitudinal trajectories of C3 in adult patients, according to the development of kidney failure; B) Subject-specific longitudinal trajectories of C3 in pediatric patients, according to the development of kidney failure.

A

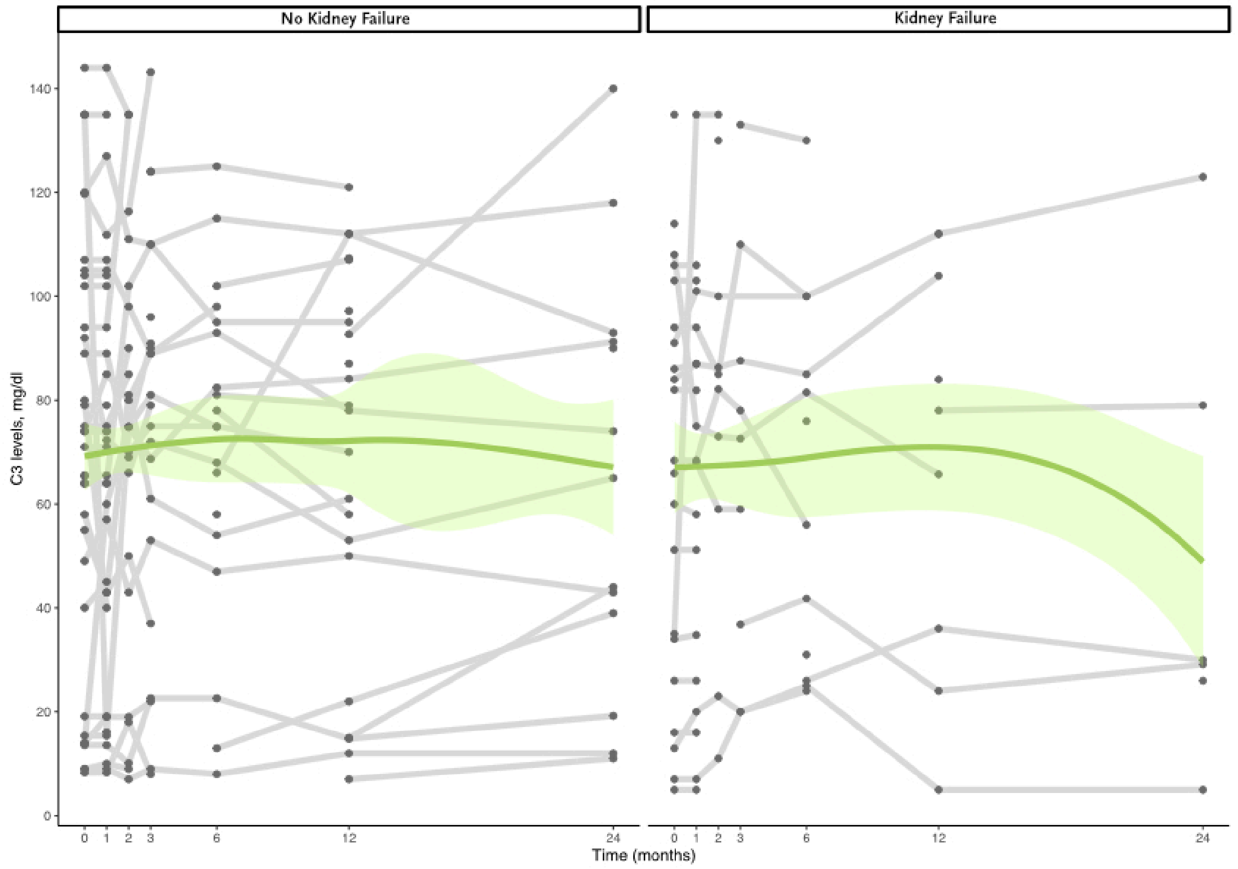

B

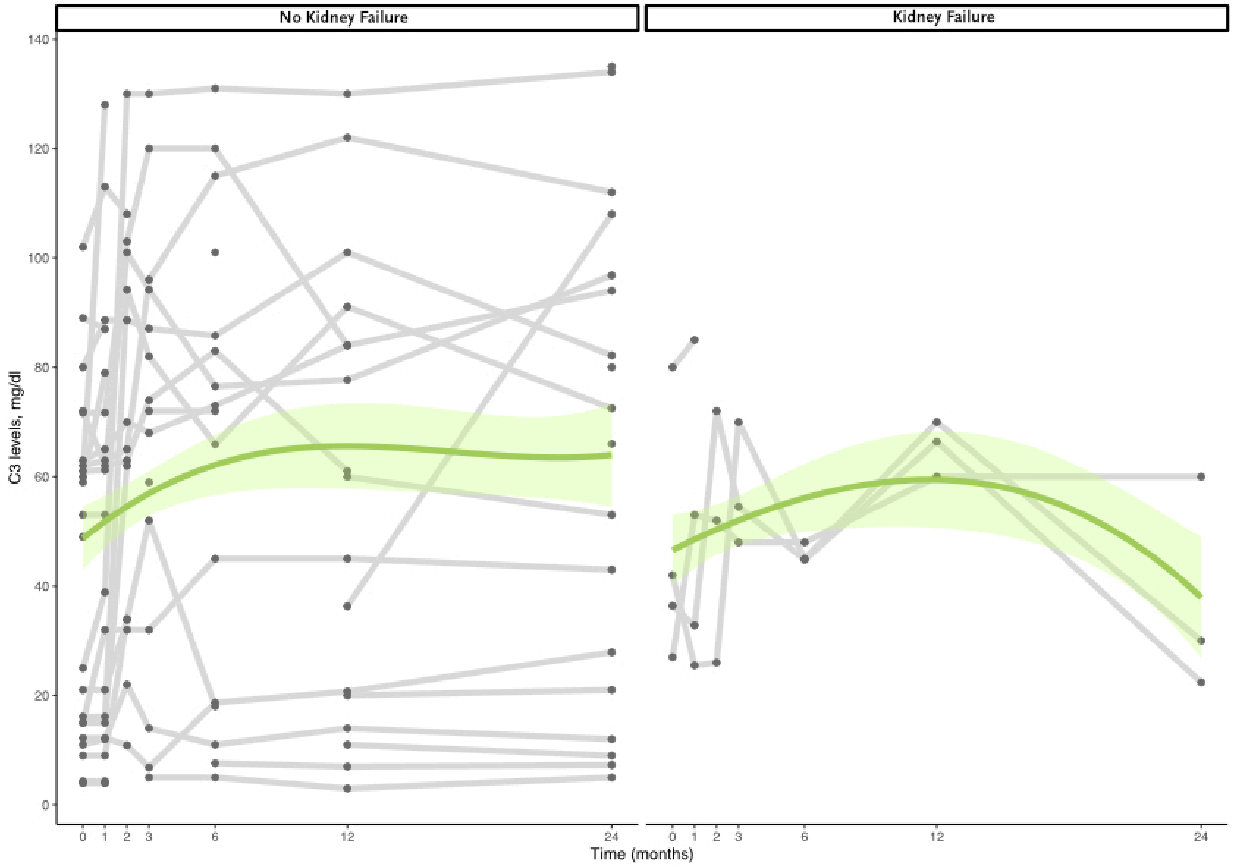

Supplementary Figure S4: Kaplan-Meier curves for kidney survival according to serum C3 levels at last follow-up.

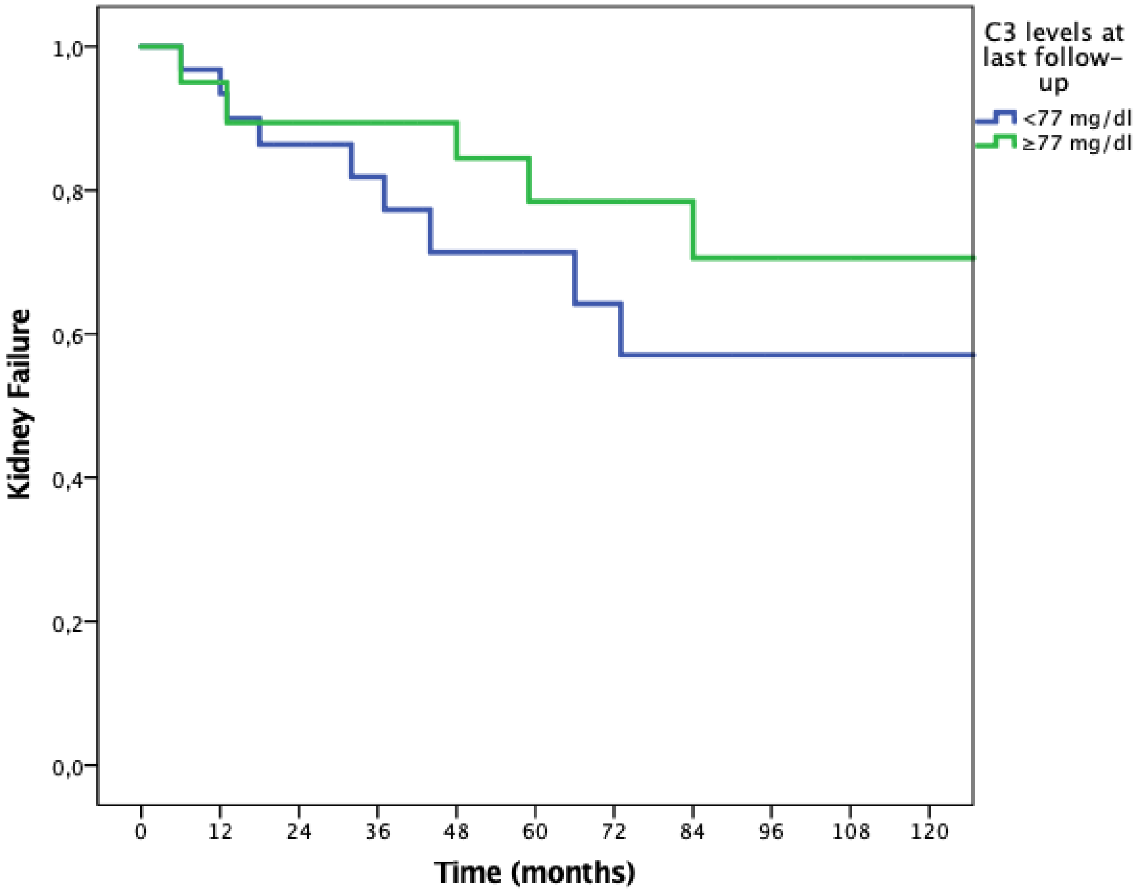

Log-Rank: 1.55; p=0.22
